# The Most Common Functional Disorders and Factors Affecting Female Pelvic Floor

**DOI:** 10.3390/life11121397

**Published:** 2021-12-14

**Authors:** Sabina Tim, Agnieszka I. Mazur-Bialy

**Affiliations:** Department of Biomechanics and Kinesiology, Faculty of Health Science, Jagiellonian University Medical College, Skawińska 8, 31-066 Krakow, Poland; sabina.tim@doctoral.uj.edu.pl

**Keywords:** pelvic floor, pelvic floor disorders, myofascial, risk factors, incontinence

## Abstract

The pelvic floor (PF) is made of muscles, ligaments, and fascia, which ensure organ statics, maintain muscle tone, and are involved in contractions. This review describes the myofascial relationships of PF with other parts of the body that determine the proper functions of PF, and also provides insight into PF disorders and the factors contributing to them. PF plays an important role in continence, pelvic support, micturition, defecation, sexual function, childbirth, and locomotion, as well as in stabilizing body posture and breathing, and cooperates with the diaphragm and postural muscles. In addition, PF associates with distant parts of the body, such as the feet and neck, through myofascial connections. Due to tissue continuity, functional disorders of muscles, ligaments, and fascia, even in the areas that are distant from PF, will lead to PF disorders, including urinary incontinence, fecal incontinence, prolapse, sexual dysfunction, and pain. Dysfunctions of PF will also affect the rest of the body.

## 1. Introduction

The pelvic floor (PF) comprises a group of dome-shaped muscles and fascia surrounding the urethra, vagina, and anus [1]. It performs many functions in our body. Through proper coordination with the nervous system, ligaments, and fascia, as well as proper contraction and relaxation of pelvic floor muscles (PFM), PF maintains the stability of internal organs and participates in continence, micturition, defecation, sexual functions, and childbirth [2]. PFM can contract voluntarily on demand and involuntarily in response to increased intra-abdominal pressure, such as during physical activity or coughing. They can also relax, returning to the initial muscle tone after voluntary contraction. Any disturbance in the functions of PFM may lead to their dysfunction [3].

This narrative review describes the anatomy and functions of PF, the myofascial connections of PF with other distant areas of the body, and pelvic floor dysfunctions (PFD) and their common causes. It is based on a search for related articles carried out in PubMed, Embase, and Google Scholar databases. The articles were selected from HI impact journals. No time limit was specified in the search. The keywords used for the search were: pelvic floor, pelvic floor dysfunction, pelvic floor disorder, fascia, myofascial, pregnancy, urinary incontinence, fecal incontinence, prolapse, aging, respiration, chronic cough, sport, physical activity, eating disorders, and sexual abuse. The review included articles that described the functions, relationships of the PF with other areas of the body, and the pathogenesis of PFD. Articles that did not describe the above dependencies, or in which their information was not complete, were excluded.

### 1.1. Anatomy of PF

**Figure 1:**
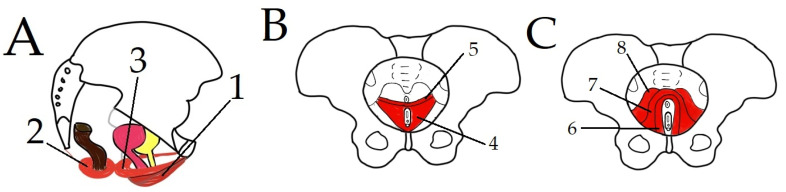
Layers of PFM: (**A**) superficial PF, (**B**) middle PF, and (**C**) deep PF. 1—Ischiocavernosus muscle; 2—sphincter ani externus muscle; 3—bulbospongiosus muscle; 4—deep transverse perineal muscle; 5—superficial transverse perineal muscle; 6—puborectalis muscle; 7—pubococcygeus; 8—iliococcygeus.

PF consists of muscles, ligaments, fascia, and the visceral system [4]. PFM can be divided into three layers [5]. The first or the most external layer is the urogenital triangle, which is composed of bulbocavernosus, ischiocavernosus, and external anal sphincter muscles. The middle layer is the urogenital diaphragm or perineal membrane, comprising superficial transverse perineal, deep transverse perineal, and sphincter urethrae [1,5]. The third or the deep layer is the pelvic diaphragm, which is made up of levator ani and coccygeus. The levator ani muscle is in turn composed of three muscles: pubococcygeus, ileococcygeuys, and puborectalis [6]. However, this division of PFM into layers is under debate, and some authors use different divisions or consider PF to be a whole entity [1]. Figure 1 illustrates the different muscles of PF.

PF can also be divided into three compartments as anterior, middle, and posterior. The anterior compartment includes the bladder and urethra; the middle compartment includes the vagina and uterus; and the posterior compartment includes the anus and rectum [7]. According to Borodni et al., there is also a fourth compartment, which includes the peritoneum composed of endopelvic fascia and perineal membrane [8].

**Figure 2:**
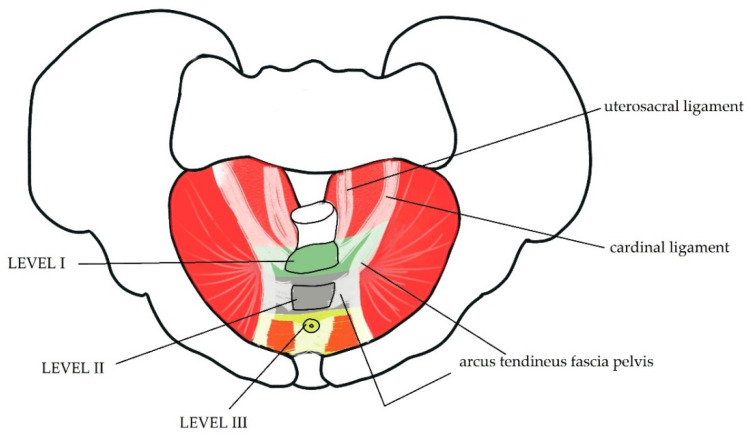
DeLancey’s three levels of vaginal support. The graphic shows a top view of PF with the levels of uterine support. The levator ani acts as an active sustaining component [10,11]. This muscle, particularly puborectalis, which is one of its parts, not only supports the vagina but also keeps the stool continent. The puborectalis muscle forms the Parks angle (Figure 3) or anorectal angle, by wrapping between the rectum and the anus [12].

The fascia and ligaments are important for maintaining the statics of the pelvic organs. The anterior and middle compartments of PF are surrounded by endopelvic fascia, a connective tissue located under the peritoneum and attached to the pelvic walls. The bladder is connected to the umbilicus by medial and lateral umbilical ligaments [9]. The uterus is also supported by numerous ligaments and muscles. Based on the location of the connective tissue, DeLancey distinguished vaginal and uterine support into three levels. Level I includes the upper part of the vagina and cervix, which is suspended by cardinal and uterosacral ligaments to the sacrum. Level II includes the middle part of the vagina which is laterally attached to the arcus tendineus fascia pelvis and arcus tendineus levator ani. Level III includes the distal or lower part of the vagina which is surrounded by the levator ani muscle and perineal body fascia [10]. Figure 2 illustrates these three levels of pelvic support.

Although numerous studies based on imaging and histology have been performed, the terminology and description of fascial PF structures remain contradictory. This is due to the differences in the examined tissue in terms of patient’s age, dysfunctions, injuries associated with childbirth and other burdens, and ethnic origin. Moreover, a majority of the studies were carried out several years ago before the recommendations for anatomical research were put forth. The most recent review describing the anatomy of PF fascia distinguishes it into perineal diaphragm, perineal body, and endopelvic fascia. The perineal membrane consists of connective tissue, mainly elastic fibers, and can be divided into anterior and posterior parts, which play different roles, but this requires a more detailed analysis. The perineal body is a pyramidal-shaped fibromuscular structure that limits the entry of the urogenital hiatus. This structure is made of collagen, adipose tissue, elastin, and smooth muscles and maintains the fascial continuity with the perineal membrane and rectovaginal fascia. Endopelvic fascia is attached to the pelvis and lines its bottom and walls. According to some authors, the endopelvic fascia continues with abdominal wall, lower back, hip, and internal obturator fasciae. Pubocervical fascia, an important but poorly described structure of PF, supports the urinary bladder, urethra, and vagina. Rectovaginal fascia, another rigid structure that supports the pelvic organs and transmits forces, is connected to the peritoneum [13].

The fascia surrounds muscles, organs, and bones and connects with various systems. It exhibits nociception and proprioception, and is essential for functional integration. The concept of fascial communication indicates that the anatomical connections of the fascia transmit pain and other effects resulting from the disorders of different parts of the musculoskeletal system. However, although the anatomy of the abdomen, lumbar region, and pelvis is well described in the literature, there are no data regarding the functional association of these structures that could explain the transmission of pain and dysfunction-related effects [14].

### 1.2. PF—Blood Supply and Innervation

Adequate blood supply and innervation are critical for proper functioning of PF. The internal pudendal artery, a branch of the internal iliac artery, is the primary artery supplying blood to PF. The internal pudendal veins drain into the internal iliac veins, while the external ones connect to the femoral vein. Deep lymphatic vessels enter the internal iliac lymph nodes. Lymphatic vessels from the genital area flow into the inguinal lymph nodes [8].

The components of the autonomic and somatic systems are involved in the complex innervation of PF. The autonomic system is further divided into sympathetic and parasympathetic system. The parasympathetic fibers that supply PF originate from 2–4 sacral nerves, while the sympathetic fibers come from the lower thoracic ganglia [6]. PFM are somatically innervated by the pudendal nerve [8].

### 1.3. Role of PF in Stabilization

PFM prevent the leakage of urine/stool by contracting and also support the lower pelvic organs. In addition, they play a significant role in stabilizing the trunk as PF is connected with abdominal muscles, diaphragm, and gluteus by myofascial continuity [8].

The pelvis acts as the site of attachment for many muscles. These muscles are not directly related to PF but are involved in pelvic girdle stabilization, maintenance of upright posture, and the movement of the trunk and limbs [15]. They also influence PFM contractions and their quality [1]. In healthy women, voluntary PFM contractions lead to the activation of abdominal and gluteal muscles. Activation of gluteal muscles as a consequence of the contraction of PFM, especially the levator ani, is attributed to the fact that these muscles are connected by the fossa ischioanalis [16]. Nevertheless, Halski et al. [17] found, that position of lower limbs influence PFM activity. In supine position, without flexion in hips and knees, PFM resting activity is the lowest, whereas functional PFM activity is the highest. Moreover, in that position, there was no influence on PFM activity by adductor magnus, rectus abdominis and gluteus maximus [17]. When the intra-abdominal pressure increases, the transverse abdominal muscle contracts with PFM, promoting anal canal closure and providing continence [18]. Rotators, mainly the internal obturator and piriformis muscle, are important for maintaining pelvic stability and PFM functionality [1,8]. The tension of the rotator cuff and gluteal muscles is primarily determined by position of the foot. However, the tension of PFM may also affect the tension and functioning of the rotators and other muscles, thus influencing the biomechanics and position of the lower limb [8].

Electromyography-based (EMG) studies suggest that the activity of PFM is determined by the position of the body. The highest activity of PFM is observed in standing position, while the activity is lowest in supine position when the legs are flexed [19]. There is also a synergistic relationship between abdominal muscles and PF. Activation of the abdominal muscles increases the activity of PFM, for example, during standing. However, when the anterior abdominal wall is relaxed in a standing position, the activity of PFM decreases [19,20]. This indicates that correct body posture and abdominal muscle tone are essential for proper PFM tension and continence [20].

### 1.4. Role of PF in Respiration

**Figure 4:**
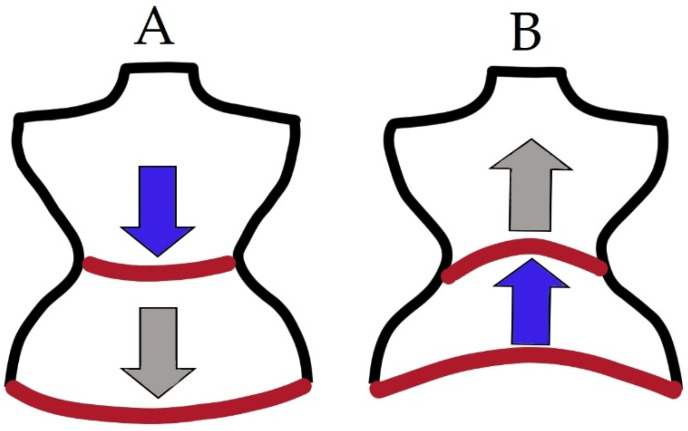
Graphic presentation of the functions of the diaphragm and PF during breathing. (**A**) The diagram shows the inhalation, concentric diaphragm function (**blue** arrow), and eccentric PFM function (**gray** arrow) (**B**).

PFM are linked by myofascial connections with the diaphragm, transverse abdominal muscle, intercostal muscles, oblique abdominal muscles, and thoracolumbar fascia, and thus, PF influences breathing [8,21]. When inhaling, the diaphragm contracts and flattens, thereby increasing the space of the thoracic cage, and moves caudally. During inspiration, the anterolateral abdominal wall slightly expands, while the PFM move posteroinferiorly. During expiration, the diaphragm relaxes and becomes cephalic, and the anterior abdominal wall muscles contract, as do the PFM, which move anterosuperiorly. The optimal intra-abdominal pressure is maintained by these cyclic tensions and muscle movements in specific directions [8,15,22]. Figure 4 illustrates the functions of the diaphragm and PFM during breathing. It is worth emphasizing that the EMG activity of PFM takes place even before the beginning of inhalation [23]. On the other hand, the amplitude of the PFM activity is higher during exhalation, due to the activation of the abdominal muscles, which also confirms the relationship between the functions of postural muscles and PFM [22]. If any of the above-described muscle parts are in constant tension, the intra-abdominal pressure will increase excessively. A chronic increase in pressure in the abdominal cavity may lead to the dysfunction of PFM or abnormalities in the respiratory pattern, resulting in further disorders [8,15,22].

The diaphragm is an essential muscle that connects PF to the structures above and below the diaphragm as well as PF to the fascia and muscles of the spine. It is attached to the xiphoid process, ribs, and the lumbar spine, where its branches reach levels L2–L3. Through ligaments, the diaphragm is connected with the lungs, heart, liver, ascending colon, and duodenum [23]. In addition, it is associated with the muscles of the abdominals, which in turn are attached to the area of the ribs, loins, hip bones, and pubic bones [21]. From a functional point of view, myofascial connections are an important component because the fascia is an active mechanical tissue, consisting of proprioceptors and nociceptors. Fascia can also change its tension, influencing the functions of the underneath muscles [24]. Moreover, respiratory functions require the support of the PF, and thus the appropriate tone of the PFM and PF ligaments is critical for ensuring proper mechanics of breathing and maintaining ideal intra-abdominal pressure [21].

A fascial connection also exists between the neck and PF. The cervical fascia arises from the neck, enters the endothoracic fascia, joins the diaphragm and subsequently the fascia transversalis surrounding the transverse abdominal muscle, reaching the end of the linea alba of the rectus abdominis muscle and finally the pubic and inguinal area, connecting the PF. The thoracolumbar fascia connects the PF with the sacrum and neck. In addition, it links the gluteal, trapezius, latissimus dorsi, and the external oblique muscles as well as the ligaments of the sacrum to the ileum [21].

Correct functioning of the fascia ensures appropriate body posture and diaphragm contractions. PF and diaphragm are connected anteriorly by transversalis fascia and posteriorly by thoracolumbar fascia. Therefore, dysfunction within any segment will affect the PF and diaphragm. On the other hand, abnormalities in the functioning of the PF or the respiratory diaphragm will affect the functioning of the fascia and muscles that are surrounded by it and internal organs [21]. PF is connected to the shoulder and pelvic girdles by the aforementioned myofascial chains and pelvic muscle attachments. Thus, it plays a key role in locomotion, positioning of the lower limbs, and load distribution from the upper limbs through the trunk to the lower limbs and vice versa [8].

Despite the controversy, it is worth highlighting the osteopathic concept of five diaphragms, which include tentorium cerebelli, tongue, thoracic outlet, diaphragm, and PF. Proper coordination of the diaphragms is necessary for efficient circulation of body fluids in the myofascia continuity. Thus, appropriate cooperation of diaphragms ensures proper nourishment and cleansing of tissues by blood and lymph. The functional problems should be approached holistically, and not by looking for dysfunction in a single area, as the body, fascia, and systems constitute an integral continuum [25].

**Figure 5:**
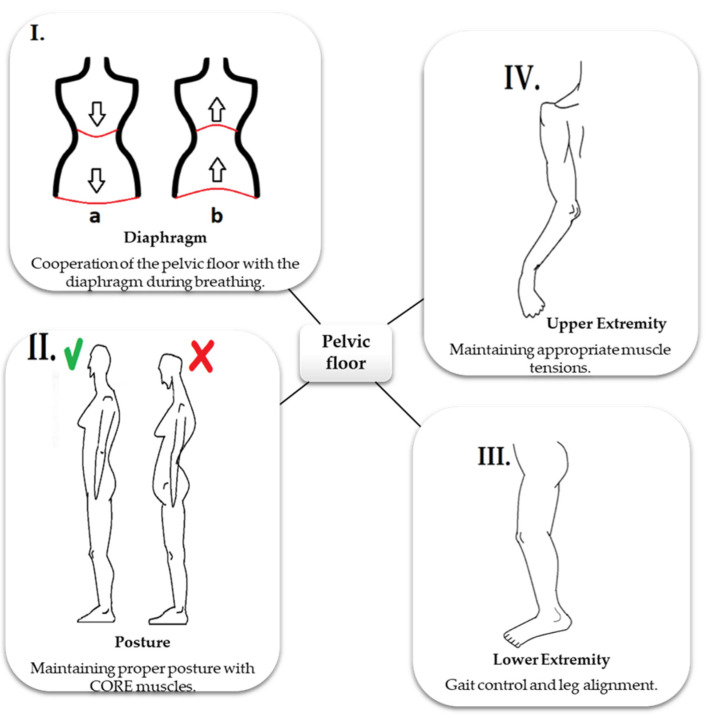
Relationship of PF with other structures and their functions. (**I**) The figure depicts the cooperation of PF with the diaphragm. PF works together with the diaphragm and takes part in respiration. During inhalation (**a**), the diaphragm descends caudally, as does PF, whereas during exhalation (**b**), the diaphragm relaxes and becomes cephalic and the PF contracts. This allows maintaining optimal intra-abdominal pressure [8,15,22]. Any disturbance in the synchronicity of the diaphragm and PF will result in pressure changes and subsequently dysfunction of other areas (e.g., disturbances in peritoneal drainage or postural stability) [21,25]. (**II**) The figure depicts the role of PF in stabilizing the body posture. PF together with postural muscles, such as abdominal, gluteal, and multifidus muscles, ensures proper stabilization. Its activity is influenced by the tension of the other core muscles. PF is also connected to the trunk by the transversalis and thoracolumbar fascia [19,20,21,22,23,24]. Myofascial disorders in this complex can change the PF tension and result in painful menstruation or intestinal disorders leading to chronic constipation. However, primary dysfunction of the PF will lead to disturbances in the entire pelvic–lumbar complex, affecting the stabilization and body posture [21,25]. (**III**,**IV**) The figure depicts that proper functioning of the PF is also influenced by the myofascial connections with the extremities. PF is connected to the lower extremity by a fascia associated with the gluteal muscles and the internal obturator muscle. These muscles control the actions of the hip joint, and thus regulate the biomechanics of the lower limb. Abnormal tension in the buttock area will disturb the movements of the hip joint, causing changes in the mechanic load on the lower limb and locomotion. Additionally, dysfunction of the hip joint and the entire lower limb will predispose to PF disorders [8,18,24]. As the gluteal muscles also play a role in postural stability, disturbances in stabilization will also lead to disorders of these muscles. PF is connected to the upper limb complex as well as the cervical spine and face through the following fascia: transversalis, mediastinal, and cervical. Therefore, disorders in this myofascial tract may lead to dysfunctions of the upper limb and diaphragm and also bruxism [8,21,25].

As mentioned earlier, PF is not only involved in micturition, defecation, organ support, sexual function, and childbirth. Through their functional connections and in synergy with other muscles and fascia, PFM aid in maintaining an upright body position, ensure balance, and participate in activities such as walking and breathing [1,21]. The functioning of PF depends on many factors, including correct myofascial tone of the entire chain system and proper functioning of the internal organs, and thus appropriate hormonal balance [21,24,25]. Figure 5 illustrates the connections of PF with other regions.

## 2. Pelvic Floor Dysfunction

PFD can occur in any compartment. Disturbances in the anterior compartment manifest as bladder problems including urinary incontinence (UI), cystocele, hesitancy, and other effects related to micturition. Dysfunction of the middle compartment negatively affects the vagina and the uterus, while that of the posterior compartment disrupts rectal functions [26]. However, as PFM, PF ligaments, and fascia are functionally connected, dysfunction in these usually affects the entire PF, while symptoms in one compartment may be more severe than in others [26]. The common causes of PFD are weakness, damage in the supporting structures, and incorrect functioning of PFM [27].

Anterior compartment disorders are primarily caused by the lowering of the anterior vaginal wall due to its pushing through the bladder and by urethral hypermobility [27]. The etiology of anterior compartment disorders is unclear, but all the proposed theories suggest that these disorders are related to the weakening of myofascial support [11]. The pubourethral ligament, which supports the urethra and the structures surrounding the anterior vaginal wall, an important element in maintaining the bladder position, is weakened and flaccid [27]. As ligaments and fascia are integrated with PFM, weakening of the fascial system will have a negative effect on the functioning of the muscles, affecting their supporting function, while incorrect PFM tension will lead to the dysfunction of the ligaments [11]. Furthermore, weakening of the fascia and supporting tissues, particularly the uterosacral and pubourethral ligaments, rectovaginal fascia, and PFM, contributes to central compartment dysfunction, which may in turn lead to the weakening of the vaginal wall and uterine prolapse [26,27]. Disturbances within the vaginal wall also affect the stability of the bladder [11]. Dysfunction of the posterior compartment results in functional defecation disorders as well as rectocele and enterocele caused by the laxity of the rectovaginal fascia [27,28]. Anorectal disorders may lead to constipation and fecal incontinence (FI) and also affect other compartments [28].

PFD can also be caused by disturbances in muscle tone and abnormal PFM contractions [27,28]. An example is the overactive PF, which is characterized by the lack of relaxation or contraction when needed, for example, during voiding or defecation [3]. PFM constantly maintains a tension to support organs, but when the voltage increases PFM becomes overactive [29]. The active (neurogenic, EMG activity) and passive components (viscoelastic properties of the myofascial tissue) are both responsible for proper tonus. Increase in tonus may occur as a protective response to inflammation in the visceral area, or if pain persists in PF for a long time [29,30]. Constant muscle tension locally impairs blood circulation and the exchange of oxygen and metabolites, leading to muscle failure and the formation of trigger points (TrPs), which refer to pressure-sensitive areas in the myofascial tissue [29]. It has been shown that the relaxation of TrPs reduces the hypertonicity of PF [30]. Conditions such as vaginismus and anismus are also caused by disorders of muscle tonus. Excessive tension in the vagina results in vaginismus, preventing penetration, tampon insertion, or gynecological examination [29]. On the other hand, anismus is caused by the lack of relaxation during defecation, which results in difficulties in stool expulsion [28].

**Figure 6:**
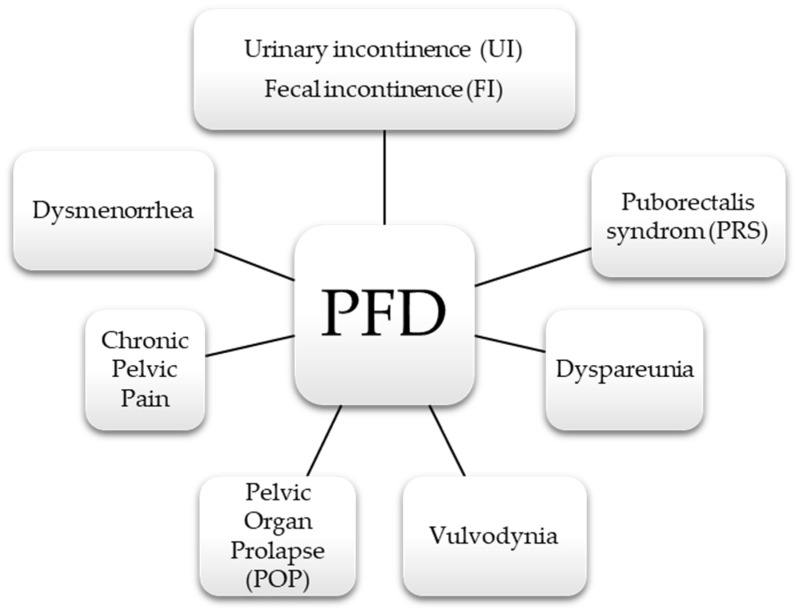
The most common PFD among women.

It is not the purpose of this review to describe each of the PFM in detail. However, in many places of this review these dysfunctions are mentioned. That is why we allow ourselves in the form of a graph to list the most common disturbances in the PF area, which shows Figure 6.

## 3. Factors Affecting PF

### 3.1. Pregnancy, Childbirth, and Postpartum

Hormonal changes during pregnancy modify the tissues of PF, negatively affecting their supporting functions. However, these changes are adopted by the female body to prepare for childbirth [31]. From the first trimester of pregnancy, the corpus luteum secretes relaxin, which indirectly participates in the degradation of elastin and collagen modification [32]. Elastin is a protein that provides resilience and elasticity to PF tissues, enabling them to return to their original shape and recoil after physical stress, while collagen provides strength and is essential for the stability of tissues [32,33]. If elastin is damaged, it may rebuild, but it will be deformed and its original properties will not be fully recovered [32]. Collagen also undergoes changes during pregnancy and is rapidly degraded by hormones [34]. Therefore, the mechanical properties of PF ligaments and PFM change, which weaken their support functions and result in PFD [31,32]. Relaxin affects not only the reproductive system and the pelvis but the whole body as well. Along with an increased level of progesterone, it causes relaxation of peripheral ligaments leading to joint instability/damage [34]. Relaxation of pelvic ligaments such as the pubic and sacroiliac joints facilitates childbirth [35].

Both VD and cesarean section reduce the strength and endurance of PFM [31]. However, the risk of PFD is higher among women with VD. When the fetal head descends, PFM are subjected to a high degree of deformation, with the generation of increased pressure, lasting up to several hours [33]. VD also causes mechanical injuries to PF and ischemia [36]. The injuries caused by VD lead to pudendal or sacral neuropathy. The longer the second stage of labor, the greater the probability of damage to PF and pudendal nerve [33]. Excessive stretching triggers the release of collagenase, which increases collagen degradation and disturbs the balance between repair and degenerative processes [37]. Moreover, the increased intra-abdominal pressure acts perpendicularly to the anterior vaginal wall, pushing it toward the levator ani, which should counteract this force. If the force exerted by the levator ani muscle is insufficient, it lowers the anterior vaginal wall and damages the uterosacral and cardinal ligaments [36]. VD also causes ligaments and fascia to detach from the bone attachments [37]. Injury to PFM and ligaments during VD impairs their support function and disrupts the ability of PFM to contract. During contraction, PFM contract inward to close the body’s orifices. On the other hand, when a muscle is damaged, the inward movement is minimal, which causes ineffective closure of the holes, leading to incontinence [38].

After delivery, the connective tissue is repaired and remodeled. Although the synthesis of collagen and elastin increases, the healed tissue will not be as strong as the old one [37]. Due to the action of hormones, which relaxed and stretched the abdominal muscles during pregnancy, an excessive increase in the distance between the rectus abdominis muscle and weakening of the anterior abdominal wall may lead to diastasis recti abdominis (DRA). Because abdominal muscles work in synergy with PF, their weakening hinder the functioning of the entire core system, including PF. Some authors have reported that a correlation exists between DRA and a reduction in the strength of PFM contraction and transverse abdominal co-contraction, as well as the occurrence of pelvic pain and impaired abdominal dynamics in response to increased intra-abdominal pressure. However, some do not confirm this relationship [39]. In postpartum women, increased muscle fatigue is observed in the lumbar–pelvic area, which negatively affects the function of PF and anterior abdominal wall. After childbirth, pelvic joints (pubic symphysis, sacroiliac joint) need time to recover from the relaxing effect. However, even after a month of delivery, the joints do not fully recover, return to their correct position, or regain their original width. Disorders within the pelvic joints disrupt their statics and may contribute to the development of lumbosacral pain [35].

### 3.2. Aging and Age-Related Hormonal Changes

The risk of PFD increases with age, especially among women. The central and peripheral nervous systems undergo degenerative changes, leading to impairment in the function, coordination, and strength of muscles [40]. The EMG activity of PFM decreases with age. Furthermore, the sensation of the bladder and anus decreases, which predisposes to UI and FI [41]. The ratio of the amount of muscle fibers to that of connective tissue decreases, as well as the thickness of the sphincter muscle layer [40]. Aging is also associated with changes in the proportion of muscle fibers: type II (fast) fibers transform into type I (slow). These changes interfere with the supporting and contracting functions of PFM [41].

In menopausal women, the levels of estrogen decrease, which affects collagen synthesis and increases tissue stiffness [42]. Estrogen receptors are also found in the smooth muscles of the bladder, urethra, vaginal mucosa, ligaments, and tissues supporting pelvic organs [42,43]. In older women, the smooth muscle fraction will be decreased in the anterior vaginal wall, which is associated with a reduced concentration of estrogens [42]. In postmenopausal women, a decreased ratio of collagen I to collagen III and V is observed. The types of collagen differ in their properties. Type I is made of the strongest fibers and is the most resistant to stretching; type III has greater flexibility; and type V is involved in wound healing. The higher the ratio of collagen I to collagen III and V, the less elastic and more stretching-resistant the tissue is. In women, PF tissues contain more collagen III which allows for childbirth. However, after menopause, the amount of collagen III increases, which reduces support functions, and as a result, the tissues become more flaccid and less resistant to loads [43].

Women are also constantly exposed to hormonal fluctuations during the menstrual cycle. Studies have shown that depending on the phase and day of the cycle, the muscle tone changes. Muscle tone is strongly related to the levels of estradiol and testosterone. Therefore, the higher the levels of these hormones on a specific day of the menstrual cycle, the better the functioning of PF [44].

### 3.3. Physical Activity

Regular physical activity can increase strength, endurance, flexibility, and composition of the body, as well as reduce the risk of many diseases [45,46]. The effect of exercise on PFD is unclear. Some studies have confirmed that physical activity can be detrimental to PF, but others did not observe this negative effect [46]. Bo et al. [46] presented two theories describing the influence of physical activity on PF. According to the first theory, PFM may be strengthened indirectly during training and the risk of symptoms such as UI, FI, and POP may be reduced, but all these may hinder labor. The second theory states that activity increases the loads on muscles, fascia, and ligaments and weakens them [47]. The negative impact of exercise seems to be associated with increased intra-abdominal pressure. Some studies suggest that intensive exercise can worsen the PF function. The incidence of urinary incontinence has been shown to be higher among women trampoline competitors, similar to professional athletes, compared to nonexercising women [45]. Trampoline jumping is very demanding for PF. During landing, a large force is exerted on PF, which must be resisted by PFM and ligaments, and PFM undergo a strong eccentric contraction. During long-term and high-intensity training, PFM cannot relax properly and are constantly in contraction, which can lead to their dysfunction in the long run. The strength of PFM contractions decreases with the duration of training [47]. However, there is no evidence proving that the activity is unfavorable to PF. Unconscious co-contraction of PFM occurs during exercise, which is actually beneficial to PF, while in some women such contraction does not occur and exercise cause excessively force on PFM and weakens them [46].

### 3.4. Obesity and Metabolic Syndrome

Excess abdominal fat (abdominal obesity) increases intra-abdominal pressure affecting PF. In women with a waist circumference greater than 80 cm, PFM, PF ligaments, and fascia are more likely to be to weakened, stretched, and stressed, which disrupt their functions [48]. Patients with metabolic syndrome also present with other clinical conditions such as abdominal obesity, hypertension, lipid disorders, insulin resistance, and diabetes [49,50]. Glycemic disturbances cause microdamage to vessels and nerves, including those in the pelvic area, affecting the sensation and functioning of the PFM and bladder [49]. Studies have shown that the greater the fluctuations in insulin levels, the worse the voluntary contractions of PFM. Moreover, metabolic syndrome increases the risk of proinflammatory states, leading to the degradation of collagen and remodeling of PF tissues [50].

### 3.5. Respiratory System Diseases

Respiratory diseases are associated with chronic cough, which contributes to the weakening of the abdominal muscles and intercostal muscles, reduces their endurance, and increases fatigue [51]. Weakening of the anterior abdominal wall will affect the stability and the biomechanics of breathing, contributing to the malfunctioning of multifidus muscles, diaphragm, and PFM. Musculoskeletal disorders lead to abnormal body posture, resulting in abdominal pressure dysregulation and poor coordination between PF and the rest of the systems [51,52]. During coughing, the diaphragm goes down and the intra-abdominal pressure increases, which will pressurize the PF and overload PFM [53].

It is also worth mentioning the effects of smoking on PF. Smoking is associated with respiratory diseases and increases the frequency of coughing. Moreover, the intra-abdominal pressure has been found to be higher in smokers than nonsmokers [52]. The nicotine contained in tobacco reduces collagen production, damages nerves, and lowers blood flow, leading to ischemia and impaired tissue regeneration in several areas of the body, including PF [53]. Local ischemia also gives rise to TrPs in PFM [29].

### 3.6. Chronic Constipation

Constipation caused by a disorder of PFM is referred to as functional bowel disease. In patients with functional constipation, the PFM (especially the anal sphincter and puborectal muscle) cannot relax during defecation, or paradoxically contract precluding defecation [54]. For proper relaxation of the levator ani, it is important to assume the correct position. Relaxation occurs at its best in the squat position, in which the hip is in the greatest flexion and the anorectal angle increases to 130°, facilitating stool expulsion [55]. Proper coordination between the abdominal muscles, PFM, and anorectal area is critical for defecation. In patients with functional constipation, an attempt to defecate will increase the tension of the abdominal muscles [56] and rectal pressure [55], while PFM may be unable to relax [56]. This increases the intra-abdominal pressure, causing straining and stretching of the PF support structures, particularly the posterior vaginal wall [57]. Disturbances in the posterior compartment may disrupt the functions of other structures of PF [28]. IBS may also be a cause of constipation. It has been shown that there is an association between IBS and chronic myofascial pain and the formation of TrPs not only in the pelvic area but also in distant muscles [58]. TrPs can increase the tonus of PFM, making defecation difficult, resulting in a vicious cycle [56].

### 3.7. Eating Disorders

The macro- and microelements supplied with food are essential for the proper functioning of skeletal muscles, including PFM. Nutrient deficiencies associated with dietary restrictions as well as intense exercise increase the risk of PFD [59]. Patients with eating disorders frequently complain of bloating, difficulty in passing stools, and PFM relaxation, and consequently constipation [60]. A weight loss below 60% impairs the endocrine system. In particular, women with anorexia suffer from hypoestrogenism, which impairs collagen synthesis and contributes to functional disorders of PFM [61]. Low-protein and low-carbohydrate diets, hypoestrogenism, and excessive exercise cause atrophy and damage the myofascial apparatus that supports the pelvic organs. This in turn affects the functioning of PF [59,61].

Bulimia, another eating disorder, also leads to PFD through induced vomiting, while excessive exercise, which increases intra-abdominal pressure, results in constipation and the need for diarrhea-inducing laxatives. Overuse of laxatives leads to abnormal changes in the colon, such as dilation, inflammation, and nerve damage, resulting in rectal prolapse. Provoked vomiting also causes an increase in intra-abdominal pressure. During vomiting, the abdominal muscles tighten to a great extent to increase the pressure in order to push the stomach contents through the mouth. Frequent vomiting significantly increases the tonus of the abdominal muscles, leading to imbalance and malfunction of the PFM, changes in their tension, and weakening of the fascia, ligaments, and the entire support apparatus [62].

### 3.8. Diseases of Connective Tissue

Abnormalities in the structure of connective tissue change its properties. Therefore, hereditary disorders of connective tissue are associated with impaired functioning of the fascia and PF ligaments [63]. Some diseases that are characterized by changes in connective tissue are Ehlers–Danlos syndrome, Marfan syndrome, and hypermobility syndrome. People with these diseases are at a higher risk of PF disorders due to improper collagen synthesis, imbalance of tissue damage and repair, and disproportion of the different types of collagen. In such patients, the amount of collagen III is higher compared to collagen I, which increases the extensibility of the tissue [64]. Connective tissue damage and disturbed repair process may also lead to the synthesis of abnormal tissue [63]. Reduced expression of collagen I and III and their decreased levels correlate with the symptoms of PFD. The PF structures are weakened if the level of elastin in the tissues is reduced, due to the increased activity of elastin-degrading enzymes [65]. Women with hypermobility are more likely to have diastasis recti abdominis after delivery. People with the symptoms of hypermobility also present with joint instability and pain and easily become tired. In addition, proprioception is impaired, and muscle control and coordination are lowered, which leads to PFM overload and myofascial disorders in these patients [64].

### 3.9. Gynecological Disorders

Patients undergoing hysterectomy experience UI, FI, and sexual dysfunction [66]. The pathogenesis of PFD will differ after surgery, and may involve damage to the nerves supplying PF structures and organs and to myofascial structures [67]. Radiotherapy, which disturbs the secretion of hormones, also has negative effects on PF [66]. Hysterectomy may lead to constipation, leading to weakening of the PF support apparatus. It also causes changes in the anatomical connections of organs and leads to compartment instability [67]. Surgery for POP is associated with the risk of PFD such as urinary incontinence, the feeling of incomplete defecation, and the feeling of a foreign body in the vagina [68]. Other factors such as age, previous births, high body mass index, and hormonal disorders also predispose to PFD after gynecological surgeries [66].

Endometriosis may also contribute to the development of PFD. Foci of endometriosis in the lumbar region disrupt the myofascial system, leading to dysfunction of PFM and disturbances in their tension [69]. TrPs often occur with endometriosis, not only in the pelvis but also in the abdomen, back, and thighs, resulting in overactivity and weakening of PFM [70]. Interesting findings have been reported by studies on women with polycystic ovary syndrome (PCOS). It appears that hyperandrogenism protects PF from weakening. Increased testosterone production occurs due to the presence of more androgen receptors, which increases the quality, strength, and endurance of skeletal muscles. On the other hand, women with PCOS encounter many problems, such as menstrual disorders and hirsutism, and are at an increased risk of metabolic and cardiovascular diseases and obesity. Studies also show that PCOS increases the risk of prolapse, suggesting that the relationship between PCOS and PFD should be investigated in detail [71].

### 3.10. Myofascial Disorders

PFD can also cause myofascial disturbances in the parts that are distant from PF but connected to it by myofascial connections [24]. Lower back pain (LBP) is often correlated with PFD [72], and may be associated with lumbar–pelvic instability and abnormal activation of core muscles. Therefore, disturbances in the functioning of the transverse abdominal muscle will affect not only PF but the stability and posture of the entire body. Women with LBP were found to have a reduced force of PFM contraction, while the activation of the transverse muscle was appropriate. This led to the conclusion that PFM has a greater influence on LBP formation compared to the abdominal muscles. It has also been shown that the function of PFM was impaired if the transverse abdominal muscle was unable to maintain its tone [72]. Abnormal movement patterns, breathing, and posture can all contribute to inappropriate load transfer resulting in PFD [73].

The asymmetry of the pelvis, which may be caused by its rotation in relation to the body axis, or the incorrect length of the muscles attached to the pelvic bones, disturbs the functioning of PFM. It also causes changes in the tension of PFM and fascia, which disrupts PFM contraction, leads to lumbar–pelvic disorders, and impairs the biomechanics of the spine and lower limbs. Pelvic asymmetry may be related to abnormalities in the distal parts of the body, for example, during the rotation of the chest or upper spine region. Restricted hip mobility disrupts the normal functioning of PF. The stability and activity of the lumbar–pelvis–hip complex, which is connected by myofascial connections and transfers forces together, are also affected by limited mobility [73].

The relationship between hip mobility and PF is also observed in the internal obturator muscle, which is closely related to PFM and the femur. Disturbances in the tension of internal obturator muscle will negatively affect PF [74]. Reduced hip joint mobility can change pelvic alignment, resulting in abnormal pelvic tilt which is compensated for by the overactivity of PFM [75]. Incorrect position of the pelvis also contributes to temporomandibular joint (TMJ) disorders. From TMJ, myofascial tension is transmitted through the spine to the pelvic girdle and PF. Therefore, TMJ disorders may also lead to PF disorders [76]. Abnormal spinal curvature is associated with excessive intra-abdominal pressure on PF, and decreased lumbar lordosis has been shown to be related to POP [77]. It was also confirmed that the activation of PFM during EMG examination depends on the position of the spine, with the highest activity observed in the neutral position and lowest in lumbar hypo- or hyperlordosis position [78].

LBP is a worldwide problem, which reduces the activity and quality of life [79]. It may be caused by the weakening of the deep muscles that stabilize the trunk, including the transverse abdominal and multilateral muscles, diaphragm, and PFM [72]. As indicated above, there exists a relationship between LBP and PFM [72,79]. Women with LBP are more likely to experience UI, and have weak PFM regardless of the occurrence of UI. On the other hand, women with both LBP and UI have weak transverse abdominal and internal oblique abdominal muscles [72]. Tenderness of PFM is also associated with LBP, which increases the resting muscle tone and decreases the ability to relax [79]. PFD associated with tension disturbances can appear as a primary or secondary response to other musculoskeletal disorders. PFM may become overactive as a chronic response to the body’s stress, as well as during abnormal toilet habits such as urinary and stool inhibition [80]. PFD can also result from restricted tissue mobility due to scars or visceral injuries [75].

### 3.11. Trauma Injuries

Another cause that can lead to PDF are pelvic injuries such as pelvic fractures. They are often caused by high-energy injuries such as vehicle collisions, or falls from heights. Traumatic injuries can damage bones, pelvic ring, blood vessels, organ, nerves, fascia, and muscles [81,82]. Pelvic fractures can lead to disruption of the bones, ligaments, and pelvic muscles, leading to instability. Common complications of pelvic fracture are chronic pain, sexual dysfunction, urinary and fecal incontinence, which may lead to anxiety and depression [81]. However, pelvic fractures occur in about 3–8% of all fractures, and unfortunately they are marked by a high mortality of up to 40% [82].

## 4. Physiotherapy and Manual Therapy

PFM exercises are the most frequently recommended for the prevention and treatment of various PFD. However, such exercises are effective only when they are performed correctly. Unfortunately, studies show that in most women with PFD proper PFM contractions do not occur [83]. Therefore, other approaches are also used with physical therapy to help patients restore normal strength, endurance, and PFM contraction. For women with weak PFM, electrostimulation and vaginal cones may be helpful to stimulate proper tension and recovery. The benefits of the applied therapy can also be confirmed by the use of biofeedback, which allows monitoring the correctness and effectiveness of PFM contractions [78]. In addition, biofeedback can increase the effectiveness of individual PFM exercises. PFM can also be activated by the magnetic field or extracorporeal magnetic innervation. This additionally stimulates the nerves in the pelvic region, not only influencing the muscles themselves but also improving the functioning of the organs [83].

On the other hand, overactivity of PFM is often associated with the formation of myofascial TrPs and can be overcome by relaxation techniques. Physiotherapists use local or global therapy to restore proper muscle tone [84]. Disrupted mobility of fascia, nerve, and joints should also be treated to restore their normal mobility and reduce pain and PFD symptoms [75]. Some relaxing techniques used include stretching, massage (deep and transverse massage), treatment of TrPs either manually or by using other devices, and dry needling. Manual relaxation of TrPs can be performed through the vagina or anus and is highly effective in improving the functions of PFM and reducing pain in these muscles. Another technique that improves PFD is the manipulation or mobilization of the hip joints, pelvis, and spine, which will help to increase their mobility and muscle recruitment [75]. Other effective PF relaxation techniques are deep breathing and global body relaxation exercises [84]. Overactivity of PFM can lead to CPP, which in turn leads to central sensitization and other comorbid psychiatric disorders. It may also cause disturbances in the hypothalamic–pituitary axis, an increase in cortisol secretion, and sensitization of nociceptive receptors. Therefore, it is important to relax both the PF area and the pelvis, as well as other myofascial connections and the nervous system [85].

## 5. Summary

**Figure 7:**
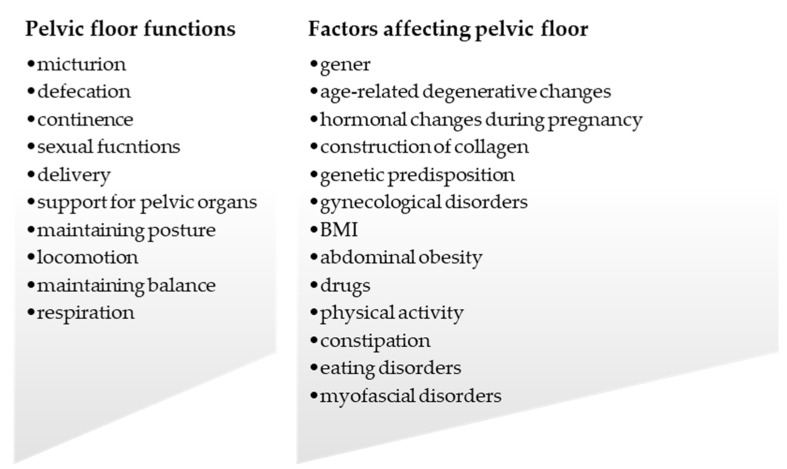
Functions of PF, and factors affecting PF.

PFD are not a marginal problem, and approximately 25% of women are affected by one of them [86]. The incidence of PFD in women increases with age. Over 50% of women aged over 80 have PFD, which affects their quality of life. The most common PFD among women is urinary incontinence [42], while about 90% of women who have overactive PFM also have PFD [80]. Many factors contribute to PFD, and the most important among them are female gender, previous births (especially VD), older age, menopause, and abdominal obesity. Chronic cough, which is observed in many respiratory disorders, constipation, and incorrect toilet habits lead to an increase in abdominal pressure, which weakens ligaments and the supporting structures of PF. Congenital disorders of the connective tissue change the properties of collagen and thus contribute to PFD. The benefits of physical activity for PFD are still debated, as the research results are contradictory exclusive. Eating disorders also lead to weakening of PF; however, this is rarely emphasized in the literature. Because the human body functions as a cohesive unit, malfunctions in one segment will have an impact on others. Through myofascial connections, abnormalities in diaphragm and breathing patterns, gait, and other musculoskeletal functions, as well as formation of TrPs lead to functional disorders in PF, which will negatively affect the rest of the body. The most important functions and factors affecting PF are shown in the Figure 7.

## Figures and Tables

**Figure 3:**
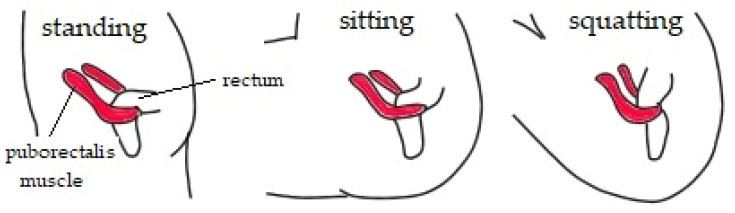
Parks angle. Depending on the position of the body, the puborectalis muscle creates different anorectal angles. In the standing position, the muscle forms an acute angle, ensuring continence, while when passing stools, the angle between the anus and rectum increases to approximately 110–130°, due to the squatting position. As shown in the graphic, the sitting position does not allow for complete relaxation of the puborectalis muscle.

## Data Availability

Not applicable.
